# MicroRNA-7-5p′s role in the O-GlcNAcylation and cancer metabolism

**DOI:** 10.1016/j.ncrna.2020.11.003

**Published:** 2020-11-10

**Authors:** Sin Yung Woo, Su Yel Lee, Seong-Lan Yu, Se Jin Park, Daeun Kang, Jin Suk Kim, In Beom Jeong, Sun Jung Kwon, Wan Jin Hwang, Chang Ryul Park, Ji Woong Son

**Affiliations:** a Department of Internal Medicine, Konyang University Hospital, South Korea; b Priority Research Center, Myunggok Research Institute, College of Medicine, Konyang University, South Korea; c Department of Nuclear Medicine, Konyang University Hospital, South Korea; d Department of Thoracic and Cardiovascular Surgery, Seoul National University Bundang Hospital, South Korea; e Ulsan University Hospital, University of Ulsan College of Medicine, South Korea

**Keywords:** Lung cancer, microRNA, Cancer metabolism, Glycosylation, OGT, PLGA, anaerobic Glycolysis

## Abstract

O-GlcNAc Transferase (OGT) is a complementary enzyme that regulates O-linked N-acetylglucosaminylation(O-GlcNAcylation) and plays a critical role in various cancer phenotypes, including invasion, migration, and metabolic reprogramming. In our previous study we found that miR-7-5p was downregulated at lung cancer cells with highly metastatic capacity. In the in-silico approach, OGT is the predicted target of miR-7-5p. To identify miR-7-5p′s role in cell growth and metabolism, we transfected various lung cancer cell lines with miR-7-5p. The expression level of miR-7-5p was confirmed by qRT-PCR in lung cancer cell lines. Western blot assays and qRT-PCR were performed to demonstrate miR-7-5p′s effect. Bioinformatic analysis indicated that OGT is a direct target of miR-7-5p. The binding sites of miR-7-5p in the OGT 3′ UTR were verified by luciferase reporter assay. To investigate the role of miR-7-5p in the cancer metabolism of non-small cell lung cancer (NSCLC) cells, mimic of miR-7-5p was transfected into NSCLC cells, and the effect of miR-7-5p on cancer metabolism was analyzed by LDH assays, glucose uptake, and mitochondrial ATP synthase inhibitor assay. O-GlcNAcylated protein level was determined by Western blot. The role of miR-7-5p in lung cancer growth was measured by MTS assays. To identify the delivery of miR-7-5p via PLGA, an *in vitro* release assay of PLGA-miR-7-5p was done. miR-7-5p was highly expressed whereas OGT showed low expression in H358, H827. However, miR-7-5p exhibited low expression while OGT had high expression in H522, H460, and H1299 cell lines. OGT were repressed by binding of miR-7a-5p to the 3′-UTR. Overexpression of miR-7-5p also diminished anaerobic glycolysis. miR-181a-5p transfection induced expression levels of OGT were diminished compared to those in the control group. O-GlcNAcylation was suppressed by miR-7-5p. Moreover, the overexpression of miR-7-5a suppressed lung cancer cell growth. miR-7-5p was released via PLGA for up to 10 days. In the present study, inhibition of OGT by miR-7-5p decreased the growth and cancer metabolism of lung cancer.

## 1 Introduction

MicroRNA (miRNA)-based therapies, either regulating the tumor suppression gene or the oncogene with miRNAs, hold great promise. However, the delivery of miRNA is a critical hurdle for clinical applications. The virus vector is a highly efficient delivery system, but its toxicity and immunogenicity limit its clinical usage [1]. Poly(lactic-co-glycolic acid) (PLGA) consists of polylactic acid (PLA) and polyglycolic acid (PGA). PLGAs are water-insoluble polymers that have excellent biocompatibility and are widely used in biomedical applications [2,3]. PLGA polymers can be readily converted into nanoparticles, which entrap DNA and RNA. PLGA nanoparticles overcome several limitations of miRNA therapy as they protect nucleic acids from degradation, achieve high-loading capacities, and afford multiple surface modifications [1,4].

O-linked N-acetylglucosamine(O-GlcNAc) is the dynamic modification of serine or threonine amino acids by a single residue of N-acetylglucosamine. Two complementary enzymes regulate this modification: O-GlcNAc transferase (OGT) and O-GlcNAcase (OGA), which add and remove sugar [5]. O-GlcNAcylation occurs in major oncogenic factors, such as MYC, and transcription factors, such as p53, NF-κB, and beta-catenin, stabilizing and increasing the activity related to tumorigenesis. O-GlcNAcylation is elevated in many types of cancers, and the OGT plays a critical role in various cancer phenotypes, including metabolic reprogramming [[6], [7], [8], [9]].

Cancer cells metabolize glucose by aerobic glycolysis, providing mechanisms to avoid immune surveillance, as well as promoting increased survival and metastasis [10]. One common characteristic of cancer cells is an increased glucose flux through the hexosamine biosynthetic pathway (HBP) and elevated UDP (uridine diphosphate) GlcNAc, which are end products of HBP. This promotes hyper O-GlcNAcylation, which explains the abundance of OGT in cancer cells. OGT plays an important role in modifying and regulating glycolytic enzymes and glucose transporters. Genetic or epigenetic changes of oncogene or tumor suppressor genes cause disturbances in the cell signaling pathway, which may generate glucose reprogramming. Targeting of metabolic reprogramming is a very promising and rapidly expanding research direction for anticancer therapy [11]. Recent studies have demonstrated that miRNAs play critical roles in glucose metabolism. In our previous studies, miR-7-5p was significantly downregulated in lung cancer cell lines with high metastatic capacity [12]. Based on this result, our team used in-silico method to find the target of miR-7-5p, and OGT is the predicted target in the in-silico approach(http://www.targetscan.org/cgi-bin/targetscan/vert_71/). We performed this study to identify miR-7-5p′s role in cell growth and metabolism for cancer treatment. We also confirmed the delivery of miR-7-5p via PLGA.

## 2 Materials and methods

### 2.1 Cell culture and growth conditions

In this experiment, we used lung cancer cell lines (HCC827, NCI–H358, NCI–H522, NCI– H1299, NCI– H460, and NCI–H226) and HeLa cell line. We purchased the HCC827, NCI–H358, NCI–H522, NCI– H1299, NCI– H460, and HeLa from the Korea Cell Line Bank, and we purchased the NCI–H226 from the American Type Culture Collection. Lung cancer cells (HCC827, NCI–H358, NCI–H522, NCI– H1299, NCI– H460, and NCI–H226) and HeLa cells were maintained in an RPMI 1640 medium (GIBCO BRL, Rockville, MD, USA) with 10% fetal bovine serum and antibiotics (100 units/mL penicillin and 100 mg/mL streptomycin).

### 2.2 TaqMan miRNA expression assay

RNA was extracted using the Accprep universal RNA Extraction Kit (BIONEER, Korea). cDNA was synthesized from the extracted RNA using the TaqMan MicroRNA Reverse Transcription Kit (applied biosystem, cat:4366597), and qPCR was performed with TaqMan PCR Master MIX (applied biosystem). The PCR equipment used was LightCycler 96 (Roche), and the dye is qPCR using FAM. and we used RNU6B for normalization. All assays were plated in triplicate.

### 2.3 PLGA nanoparticle synthesis

We left the PLGA particles, kept at −20° Celsius, in room air for 30 min (PLGA: Corbion Purac: PURA SORB PDLG 5002A). Stock solution was made by adding 540 μL of D.W. to microRNA (negative control- 50 nmol of miR-7-5p in one tube) ordered from Genolution. We transferred microRNA and supermidine to 1 mL and 5 mL tubes respectively and incubated them in the RT for 30 min. We inserted 20 mg of PLGA into two 20-mL tubes each. Micro RNA and 400 μL of diluted Spermidine were added to each tube after adding 5 mL of dichloromethane. The tubes were sonicated at 30 for 1 min. We inserted 10 mL of PLGA into 1% WT PVA, and a second sonication was done at 65 for 20 s. The tubes containing PLGA were left with the lid open under a dry hood and were dried for 24 h. We transferred the PLGA into two 2-mL tubes and freeze dried them using a SpeedVac. The freeze-dried sample was kept in the freezer at −20° Celsius.

### *2.4* qRT-PCR

Total RNA was isolated with TRIzol solution (Ambion) according to the manufacturer's protocols. The first strand of cDNA was synthesized by using the oligo (dT) primer system (Super- Script III First-strand Synthesis System; Invitrogen). Aliquots of the reaction mixture were used for the qPCR amplification with the CFX96 system (Bio-Rad Laboratories, Hercules, CA, USA) using iQ SYBR Green Supermix (Bio-Rad Laboratories). The PCR was run for 40 cycles of denaturation at 95 °C for 15 s, annealing at 56 °C for 15 s, and elongation at 72 °C for 15 s. Gene expression was quantified by the comparative CT method, with normalization of CT values to the ß-actin housekeeping gene. After amplification, melting curve analysis was performed to ensure the specificity of the products.

### *2.5* Western blot

OGT: H460 cells were lysed in Pro-Prep protein extraction solution (INtRON Biotechnology, Gyeonnggi-do, Korea), miR-7-5p transfection after 72 h. An equal amount of protein was dissolved in 8% SDS-PAGE gels (Laemmli, 1970). The primary antibodies used for the analysis were mouse anti-OGT (1:1000 cell signaling) and b-actin antibodies (1:2000; Santa Cruz Biotechnology)

O-GlcNAc: HeLa cells were lysed in Pro-Prep protein extraction solution (INtRON Biotechnology, Gyeonnggi-do, Korea). The miR-7-5p was transfected after 72 h. The Mini Gel Tank System (Invitrogen) was used for protein sample gel loading, and an iBlot2 (Invitrogen) membrane transfer was done. The primary antibodies used for the analysis were mouse anti- O-GlcNAc (1:1000 cell signaling) and b-actin antibodies (1:2000; Santa Cruz Biotechnology)

### *2.6* Luciferase reporter assays

To verify that miR-7-5p can regulate OGT gene directly, we generated a Renilla luciferase reporter plasmid cloned downstream to a segment of the 3′UTR containing putative miR-7-5p binding sequences, which predicted the binding site, position 268–275 bp of OGT 3′UTR (NM_181672) using microRNA.org. The constructs were then transfected into NCI–H1299 cells with miR-7-5p mimic, mimic-negative control, and Renilla-Firefly reporter plasmid. After 48 h, the Renilla-Firefly luciferase activity was measured using a Lumat LB9501 instrument (Berthold, Bad Wildbad, Germany), and the results were normalized using the activity of the Firefly luciferase. All experiments were performed in triplicate.

### 2.7 Glucose-uptake assay

We transfected H460 with a miRNA -7-5p mimic (Genolution) and a miRNA mimic-negative control #1 (Genolution). After 48 h of transfection, the rate of glucose uptake was measured, using the Glucose Uptake Assay Kit Colorimetric (Abcam, Cambridge, UK) according to the manufacturer's instructions.

### 2.8 Lactate dehydrogenase assay

After 72 h of transfection with miRNA -7-5p mimic (Genolution) and miRNA mimic-negative control #1 (Genolution) in H1299 cells, the CytoTox 96® Non-Radioactive Cytotoxicity Assay (Promega, Madison, USA) was used to measure quantitative lactate dehydrogenase, (LDH) according to the manufacturer's instructions.

### 2.9 Mitochondrial ATP synthase inhibition assay

Following miRNA transfection on day three, H1299 cells were treated with oligomycin A for 30 min. Since then the cellular ATP changes were measured by the following procedure with the CellTiter-Glo® Luminescent Cell Viability Assay (Promega) according to the manufacturer's instructions. We seeded the cells into 96 well plates at 5000 cells/well in 200 μL of media/well. After treatment of the compound, we incubated the plates at 37 with 5% CO2 for 24 h. We added a volume of CellTiter-Glo® 2.0 Reagent equal to the volume of cell culture medium present in each well, mixed the contents for 2 min on an orbital shaker to induce cell lysis to stabilize the luminescent signal, and incubated the plates for 10 min at room temperature. We read the luminescence by a GloMax® Discover Microplate Reader (Promega).

### 2.10 MTS assay

H1299 cells, 3 X 10^3^, (Promega, CellTiter 96® AQueous One Solution Cell Proliferation Assay, G3582) were seeded into a 96-well plate. We added 20 μL of the CellTiter 96® AQueous One Solution Reagent to the cells in 100 μL of culture medium. The mixture was incubated at 37 °C for 2 h. It then was measured for an optical density value at 490 nm.

### 2.11 Statistical analyses

The statistical differences between the two groups were analyzed by using the Student's t-test. We considered the differences to be statistically significant at *P* < 0.05.

## 3 Results

### 3.1 miR-7-5p regulates OGT

**Fig. 1:**
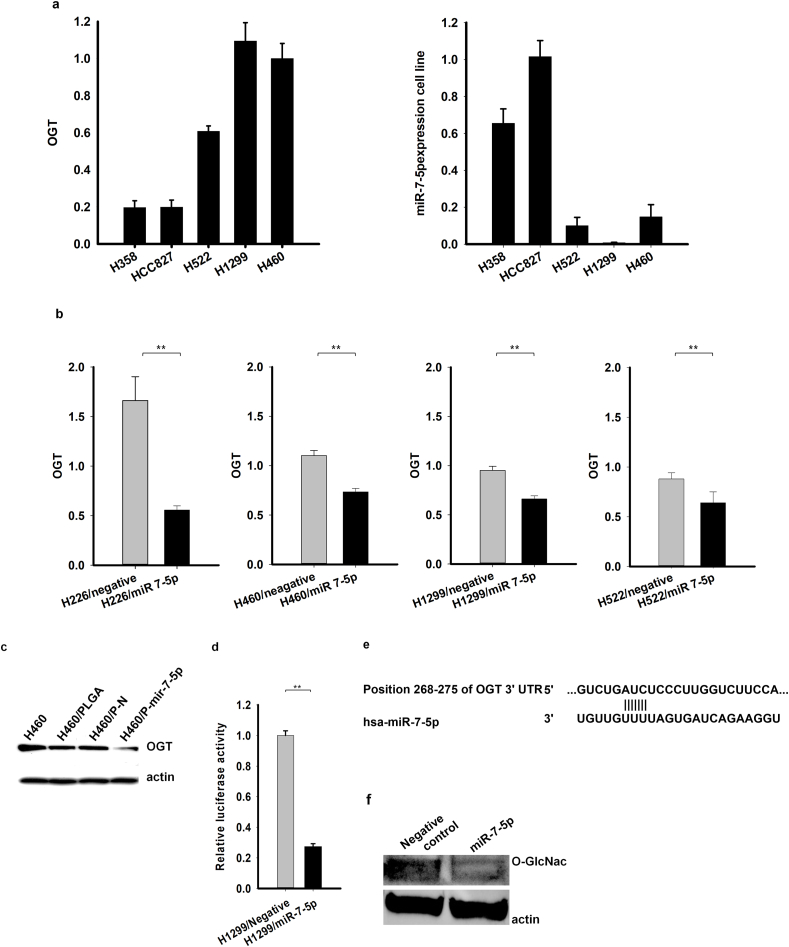
miR-7-5p regulated OGT. (**a):** H1299 and H460 exhibited the highest expression of OGT and the lowest expression of miR-7-5p. **(b):** qRT-PCR analysis shows downregulation of OGT expression after miR-7-5p transfection in the cell lines H226, H522, H1299, and H460. (c): Western blot shows that OGT protein is downregulated after miR-7-5p transfection. Beta-Actin was used as an internal control. (**d):** miR-7-5p transfection reduced the luciferase activity of OGT 3′UTR. **P < 0.01. **(e):** The schematic diagram of miR-7-5p binding sites in the 3′-untranslated regions (UTR) of OGT (**f):** O-GlcNAcylated proteins transfected with miR-7-5p were downregulated.

We examined the relationship between miR-7-5p, a well-known tumor suppressor, and OGT, a notable enzyme involved in anaerobic glycolysis of cancer cells. Cell lines with low miR-7-5p expression (H522, H1299, H460) had a higher expression of OGT, whereas cell lines with a high miR-7-5p expression (H358, HCC827) had a lower expression of OGT (Fig. 1a). We did a qRT-PCR analysis for the mRNA level in the H226, H460, H1299, and H522 cell lines to measure the OGT expression following miR-7-5p transfection*.* We found that miR-7-5p significantly reduced the OGT level in the H226, H460, H1299, and H522 cells (Fig. 1b), demonstrating that OGT is the target gene of miR-7-5p. To measure the effect of miR-7-5p on the protein level, a Western blot analysis was done. As shown in Fig. 1c, the OGT protein was downregulated following miR-7-5p transfection*.* To verify that miR-7-5p regulates OGT directly, a luciferase reporter assay was performed. Renilla activity was significantly lower in the miR-7-5p mimic compared to the miRNA negative group (Fig. 1d and e).

### 3.2 miR-7-5p inhibits O-GlcNAcylation

To confirm the inhibitory effect of miR-7-5p on the O-GlcNAcylated protein level, we performed a Western blot analysis in HeLa cell line using primary antibody O-GlcNAc (mouse-Cell Signaling) and secondary antibody goat anti-mouse IgM (Santa Cruz). As a result of the Western blot, the O-GlcNAcylated proteins in cancer cells transfected with miR-7-5p were downregulated. (Fig. 1f). Thus, we could confirm that miR-7-5p downregulates O-GlcNAcylated on the protein level.

### 3.3 miR-7-5p regulates cancer cell metabolism

**Fig. 2:**
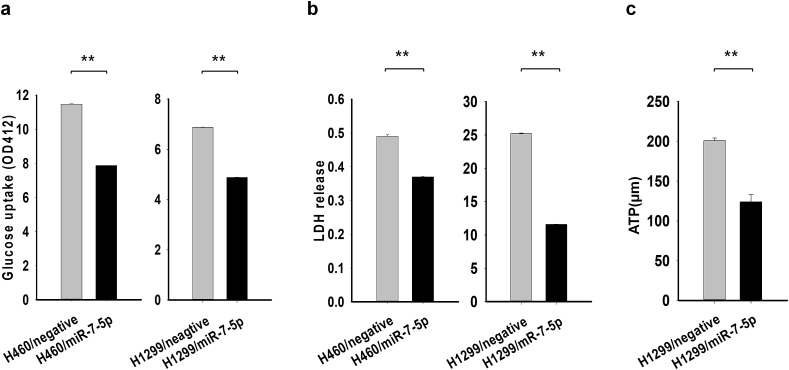
miR-7-5p regulates cancer cell metabolism. (**a):** H460 transfected with miR-7-5p showed a decrease in glucose uptake. **(b):** Decreased LDH release after miR-7-5p transfection in the cell line H1299. (**c):** ATP production in cancer cells is markedly decreased after miR-7-5p transfection. **P < 0.01.

To investigate the inhibitory effect of miR-7-5p on cancer cell metabolism, we did an LDH assay, a glucose uptake, and a mitochondrial ATP synthase inhibitor assay. As shown in Fig. 2a, the glucose uptake was decreased in cancer cells transfected with miR-7-5p. Cancers cells transfected with miR-7-5p showed a decrease in LDH release (Fig. 2b). Lastly, we investigated the effect of miR-7-5p in ATP production within anaerobic glycolysis. Cancer cells were treated with oligomycin A to eliminate oxidative respiration. Decreased ATP production in H1299 cells are transfected with miR-7-5p shows that miR-7-5p plays a significant role in anaerobic glycolysis (Fig. 2c).

### 3.4 miR-7-5p inhibits cell growth

**Fig. 3:**
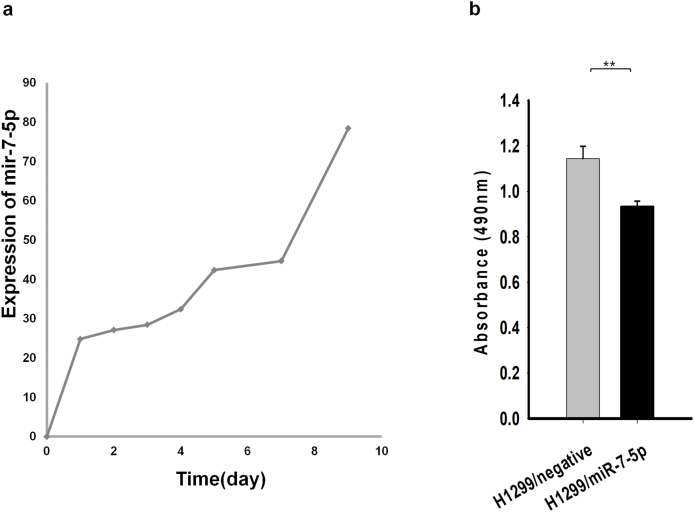
**(a):** miR-7-5p was released for up to 10 days via PLGA. (**b):** H1299 cells transfected with miR-7-5p had lower viability.

To confirm the delivery of miR-7-5p via PLGA, an *in vitro* release assay of PLGA-miR-7-5p was done. miR-7-5p was released via PLGA for up to 10 days (Fig. 3a). We transfected the H1299 cells with miR-7-5p for 48 h and assessed their viability by using an MTS assay. The miR-7-5p transfection resulted in an approximately 20% decrease in the viability of the H1299 cell line (Fig. 3b). Thus, it could be concluded that miR-7-5p inhibits cell growth.

## 4 Discussion

In this study, we observed substantial OGT suppression by miR-7-5p in lung cancer cell lines. The expression of miR-7-5p inhibited OGT activity by binding to the OGT 3′-untranslated region (UTR). Moreover, OGT was confirmed as a direct target of miR-7-5p via the luciferase reporter assay. In conclusion, miR-7-5p functions as a tumor suppressor by targeting OGT, thus inhibiting glycolysis and metabolism in lung cancer.

MicroRNAs are small, non-coding RNA molecules of 18–24 nucleotides that regulate gene expression, transcribed by RNA polymerases II and III. In human cancer, miRNA expression is dysregulated, functioning as either oncogenes or tumor suppressors Amplification or deletion of the miRNA genes, abnormal transcription of miRNAs, and defective miRNA synthesis are some of the ways in which miRNAs are dysregulated in cancer cells. Such cancer cells with altered miRNA expression generate proliferation, evade growth suppressors, resist apoptosis, activate invasions and metastasis, and induce angiogenesis [13]. Thus, identifying the critical targets of miRNAs and regulating the associated miRNAs accordingly in cancer cells show great promise as a cancer treatment [14].

Until the mid-1980s, glycoprotein was thought to be nonexistent in the nucleus or the cytoplasm. However, thousands of O-GlcNAcylated proteins are founded in the nucleus, cytoplasm, and mitochondria. O-GlcNAcylation is the process of adding O-GlcNAc via an O-linkage to serine or threonine residues of intracellular proteins. This modification increases enzyme activity, as well as interfering with protein stability and interacting to affect protein half-life. O-GlcNAcylation can alter fundamental cellular processes, such as transcription, epigenetic modifications, and cell signaling dynamics [15,16]. O-GlcNAc transferase adds O-GlcNAc onto the free hydroxyl of select serine and threonine residues of the target proteins. Recent studies report that the level of O-GlcNAc and OGT increases in tumor tissues as compared to adjacent healthy tissues. Increased O-GlcNAcylation contributes to increased tumor cell proliferation, tumor formation, invasion, and metastasis. O-GlcNAcylation could enhance the anchorage-independent growth and invasion of lung cancer cells [17,18]. In the present study, inhibition of OGT by miRNA decreased lung cancer metabolism.

**Fig. 4:**
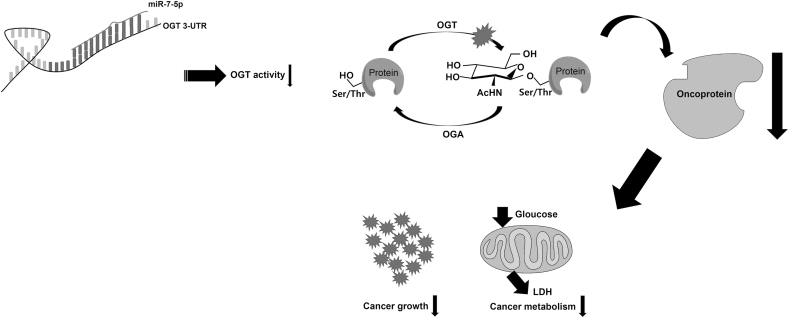
A schema of the hypothesis. miR-7-5p suppressed the activity of OGT by binding to the 3-UTR site of OGT, inhibiting the O-GlcNAcylation of oncoproteins. It could also regulate glucose metabolism through Akt, c-Myc, ChREBP, NF-κB, and HIF-1. Consequently, miR-7-5p inhibits cancer metabolism and the growth of cancer cells.

Cancer cells supply many nutrients required for rapid cell proliferation, to produce energy, and to increase the biosynthetic activity of various cellular constituents required for cell division. The reprograming of glucose metabolism and lipid metabolism within the tumor microenvironment is an effective strategic shifting for survival and invasion [19]. Accumulating evidence suggests that OGT may act as a nutrient sensor that links hexosamine biosynthesis pathway to oncogenic signaling and the regulation of glucose and lipid metabolism. OGT affects gene expressions involved in cellular metabolism through histone modifications and assembly of gene transcription complexes [20]. Akt, c-Myc, ChREBP, NF-κB, and HIF-1 that affect metabolism have been found to be tightly associated with O-GlcNAcylation [21,22]. In addition, O-GlcNAcylation directly regulates glucose metabolism by affecting the activity of phosphofructokinase 1 (PFK1) [23]. Glucose ﬂux through the glycolytic pathway can be diverted into the pentose phosphate pathway (PPP), which plays a vital role in anabolic biosynthesis and antioxidative defense. Glucose-6-phosphate dehydrogenase (G6PD) is a rate-limiting enzyme in the pentose PPP. G6PD is dynamically modiﬁed by O-GlcNAc and glycosylated in lung cancers [8]. Our hypothesis is illustrated in Fig. 4.

The diversity of adenocarcinomas as compared to squamous cell carcinomas (SCCs) warrants a tailored treatment, according to the different types of lung cancer. Adenocarcinomas have more EGFR mutations than do SCCs, and thus there are more options for targeted therapy, as compared to SCCs [24]. According to one study that conducted molecular profiling, there are 18 statistically significant mutations in adenocarcinoma, whereas there are 11 mutations in squamous cell cacinoma [25]. In a study comparing the PET CT characteristics of different histologic types of lung cancer, adenocarcinomas have differing standard uptake values (SUVs), according to the level of tumor differentiation. Poorly-differentiated adenocarcinomas have higher SUVs as compared to well-differentiated adenocarcinomas of the same size. However, in squamous cell carcinomas, the SUVs do not differ according to the level of tumor differentiation [26]. Different types of lung cancer have different levels of metabolism; thus, treatment may be tailored according to the differing levels of metabolism.

## 5 Conclusions

In summary, our study demonstrates that miR-7-5p can reprogram cancer metabolism and.

O-GlcNAcylation in NSCLC. We identified miR-7-5p as regulator of OGT expression. Therefore, miR-7-5p appears to represent a novel therapeutic candidate to alleviate metabolically active lung cancer.

## Funding

This study was supported by a 10.13039/501100003725National Research Foundation of Korea Grant funded by the Korean Government (NRF-2018R1D1A3B07048311)

## Contributions

Designed research (project conception, development of overall research plan, and study oversight): SJW, CRP; conducted research (hands-on conduct of the experiments and data collection): SYL, SYW, SLY; wrote paper: SYW, SYL, SLY, SJW, CRP. All authors read and approved the final manuscript.

## Ethics declarations

This study was conducted using cell lines, and there is no ethical problem since the experiment was not conducted using humans or animals.

## Consent for publication

All authors of this study agreed to publication.

## CRediT authorship contribution statement

**Sin Yung Woo:** Writing - original draft, Writing - review & editing. **Su Yel Lee:** Writing - original draft, Writing - review & editing, Methodology. **Seong-Lan Yu:** Writing - review & editing, Methodology. **Se Jin Park:** Writing - review & editing, Data curation, Software. **Daeun Kang:** Writing - review & editing, Methodology. **Jin Suk Kim:** Writing - review & editing. **In Beom Jeong:** Writing - review & editing, Investigation. **Sun Jung Kwon:** Writing - review & editing, Investigation. **Wan Jin Hwang:** Writing - review & editing, Conceptualization, Writing - review & editing, Supervision. **Ji Woong Son:** Conceptualization, Writing - review & editing, Funding acquisition, Supervision.

## Declaration of competing interest

The authors declare that they have no conflicts of interest.
